# Grand Challenges in Infectious Diseases: Are We Prepared for Worst-Case Scenarios?

**DOI:** 10.3389/fmicb.2020.613383

**Published:** 2020-11-30

**Authors:** Axel Cloeckaert, Karl Kuchler

**Affiliations:** ^1^INRAE, UMR ISP, Université de Tours, Nouzilly, France; ^2^Department of Medical Biochemistry, Max Perutz Labs Vienna, Medical University of Vienna, Vienna, Austria

**Keywords:** infectious diseases, mortality, antimicrobial multidrug resistance, host-pathogen interactions, research strategies, vaccines, COVID-19, polymicrobial disease

## Introduction

**Figure 1:**
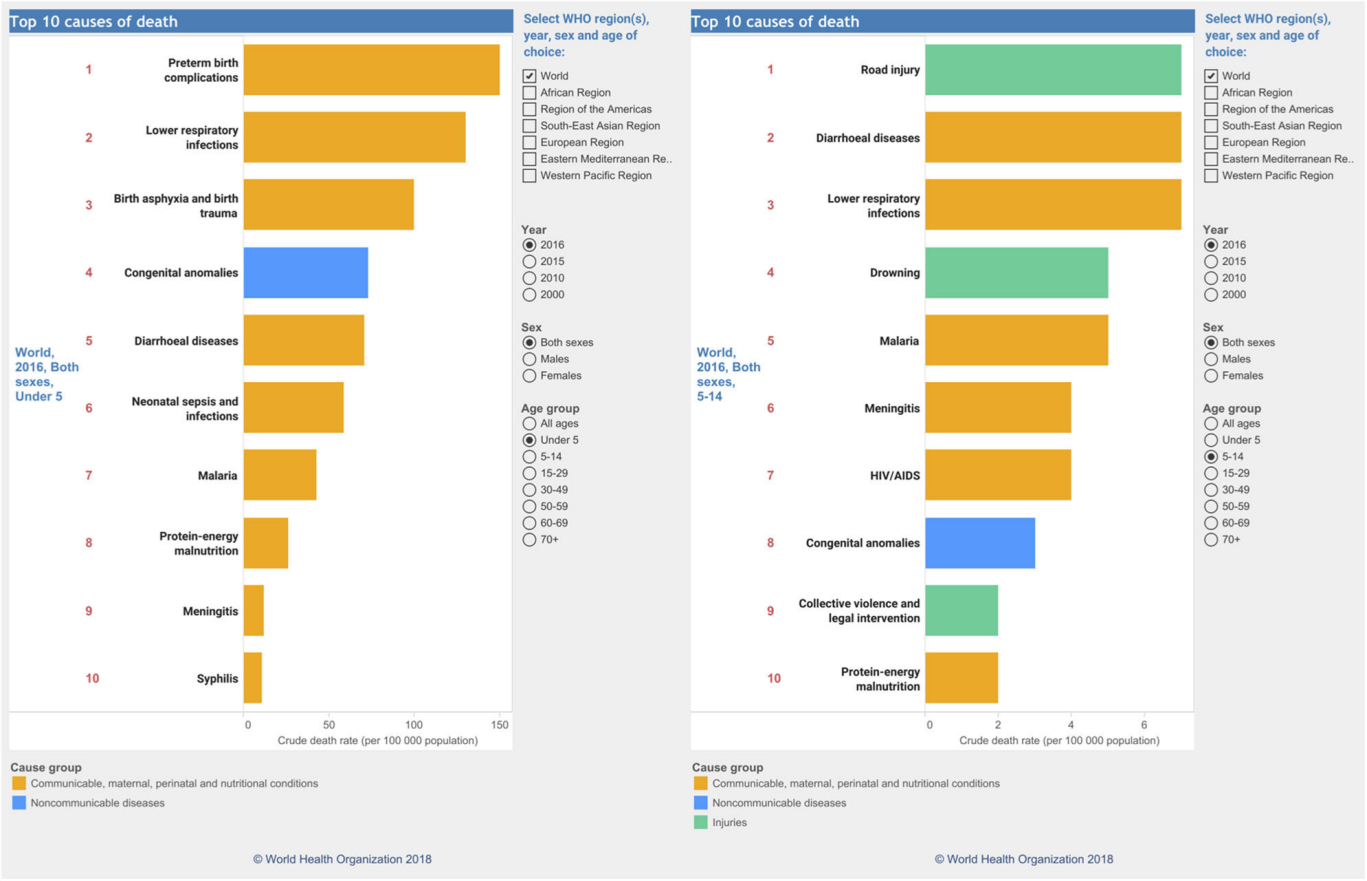
Global causes of childhood deaths according to the WHO. Causes of deaths for the year 2016 in children from 0–5 in age (left) and 5–14 (right). Data were taken and reproduced with kind permission from the website of the World Health Organization (https://www.who.int/data/gho/data/themes/topics/causes-of-death).

The serendipitous discovery of *antibiotics* by Fleming (1946), the groundbreaking work on *vaccination* by Robert Koch and Louis Pasteur, and the genesis of *prophylactic vaccination* (https://www.historyofvaccines.org/timeline/all) were undoubtedly the most breath-taking medical discoveries during the past two centuries. Anti-infective drugs and vaccines, along with tailored therapies by personalized precision medicine for many cancers and circulatory diseases, as well as access to high-end medical technologies, have been saving uncounted millions of human lives and increased longevity. Despite all, and this is deeply concerning, infectious diseases (Morens et al., 2004) stand out by far as the single major causes of childhood mortality until the age of 14 all over the world (Figure 1). Across all age groups, infectious disease deaths similar to cardiovascular diseases (https://www.who.int/data/gho/data/themes/topics/causes-of-death).

Less-privileged countries are held hostage by deadly pathogens, causing malaria, diarrhea, Ebola, meningitis, tuberculosis, HIV/AIDS, and many other viral, parasitic and fungal infections. The recent dramatic re-emergence of almost forgotten measles even in developed countries also demonstrates that health authorities have been inefficient in raising awareness about safety of preventive and prophylactic vaccination (Hotez et al., 2020). Two hundred years of success in *prophylactic vaccination* provide a compelling argument about benefits. Vaccines eradicated diseases like polio, hepatitis, diphteria, meningitis, measles in most developed countries (Poland and Barrett, 2009). Despite irrational and dangerously erupting anti-vaccine movements that fuel the dwindling public confidence (Hussain et al., 2018), therapeutic vaccines have also been effective at the intersection of infections and cancer (Drolet et al., 2015), as shown by the successful human papilloma virus vaccine (Schiller and Muller, 2015). However, screeming and screeching anti-vaxxer movements have often been louder than scientific facts, and showcased how abusive “*alternative facts*” communication strategies can influence even educated minds, sometimes regressing medicine back into medieval ages (Hussain et al., 2018). Importantly, traditional and new vaccination technologies (Wimmers and Pulendran, 2020) will be required to establish herd immunity (Randolph and Barreiro, 2020), a pivotal road block preventing the expansion of local infectious diseases into global pandemics.

The same applies to anti-infective drug pipelines although this has become a challenge. The increasing overuse and misuse of antimicrobials, as well as environmental disposal of millions of tons of *antibiotics, fungicides, herbicides* and *pesticides* from agriculture and animal mass production, has been selecting for multidrug-resistant pathogens that emerge at astonishingly fast paces, often outcompeting the time required for generating new antibiotics. Hence, pronounced and (re)emerging multidrug resistance (MDR), as most evident in the ESKAPE-pathogens (*E. faecium, S. aureus, K. pneumoniae, A. baumannii, P. aeruginosa, Enterobacter* spp.) poses major obstacles to anti-infective therapies (De Oliveira et al., 2020), including in fungal pathogens (Perlin et al., 2017; Berman and Krysan, 2020) and specifically in the emerging fungus *Candida auris* (Meis and Chowdhary, 2018). The WHO put global antimicrobial resistance on the radar screens years ago (https://www.who.int/en/news-room/fact-sheets/detail/antimicrobial-resistance), and governments have now recognized the relevance of MDR, as reflected in recent G20 summit statements (https://www.gesundheitsforschung-bmbf.de/en/GlobalAMRHub.php). While politics keep emphasizing the need to fight MDR microbes and to combat pandemics, promises remain promises and meaningful actions often fall victim to shortage of funds or erratic political decisions that ignore the scientific facts.

**Figure 2:**
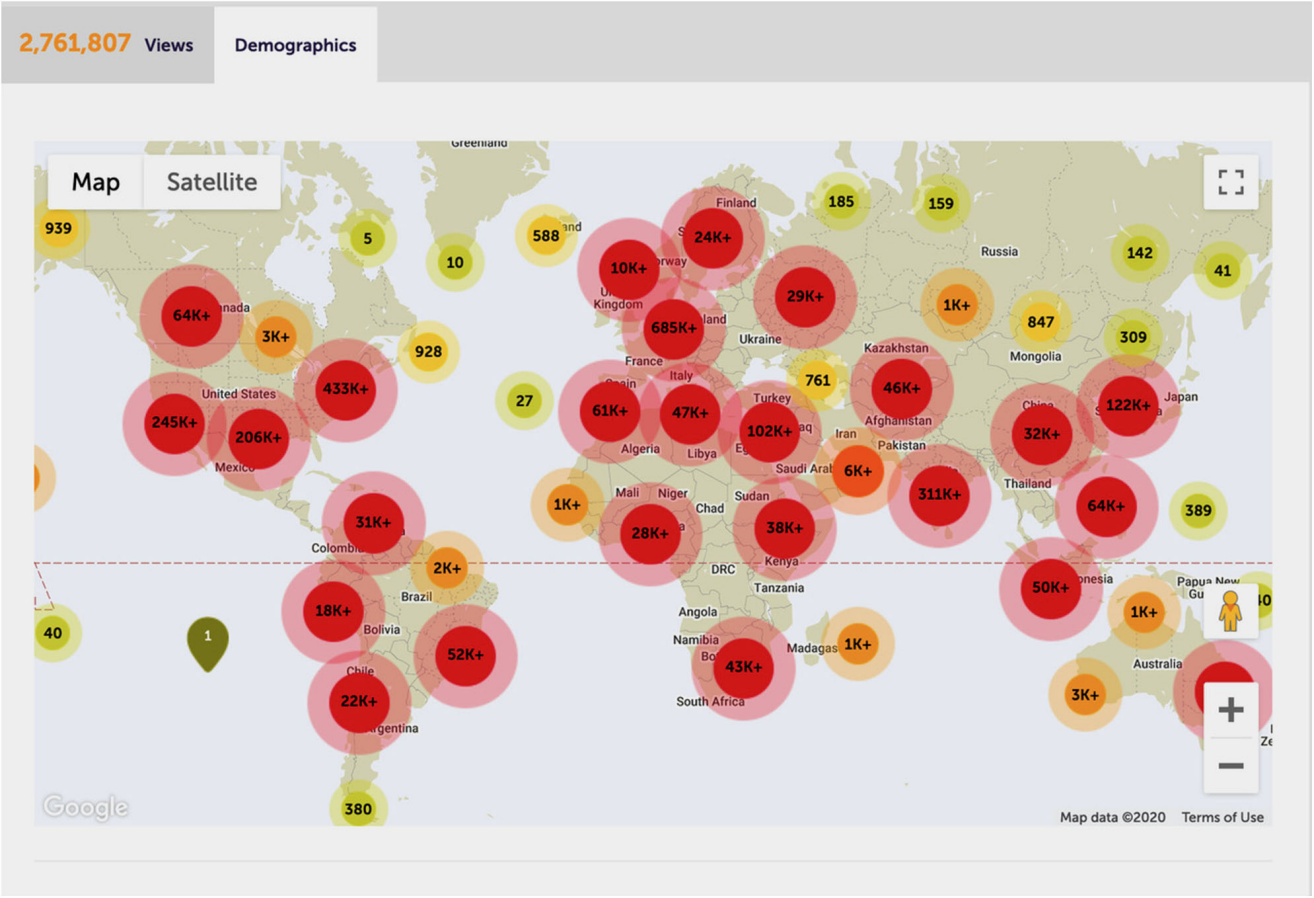
The most-viewed COVID-19 Frontiers research topic. Global impact and demographics of the most-viewed Frontiers research topic on COVID-19 (https://www.frontiersin.org/research-topics/13377/coronavirus-disease-covid-19-pathophysiology-epidemiology-clinical-management-and-public-health-resp#impact), overall attracting about 2.8 million views as per 1 October 2020. With permission of Frontiers. Data and carton was reproduced from the Frontiers website.

Strikingly, SARS-CoV-2 causing COVID-19, despite its early discovery and alerts about its pandemic potential (Cao et al., 2020), has mercilessly shown that even healthcare systems in developed countries are ill-prepared to cope with rapidly emerging pandemics, especially when treatments are unavailable and disease phenotypes are pleiotropic (Tang et al., 2020). We should have known better and the world should have been better prepared after HIV/AIDS or the Spanish Flu, the latter killing an estimated 50–100 million people at the onset of the 20th century (Gambotto et al., 2008). This explains why the COVID-19 pandemics has been receiving unprecedented global attention. Remarkably, some 282 *Frontiers Research Topics* so far addressed COVID-19 (https://www.frontiersin.org/research-topics?domain=all&query=covid-19), with the topic “*Coronavirus Disease (COVID-19) Pathophysiology, Epidemiology, Clinical Management and Public Health Response*” alone attracting almost ~2.8 million views (Figure 2).

Still today, most countries lack pandemic emergency plans following science-based governance. However, the evolution and adaptive co-evolution of viral and microbial as well as zoonotic pathogens affecting the human host (Woolhouse et al., 2002) makes pandemics of high dynamics possible at any given time. Hence, delayed or inappropriate responses as in part for COVID-19, has been contributing to a dramatic loss of human lives, as well as unprecedented damage to economies, healthcare and education systems. The tremendous socio-economic impact pandemics pose for worldwide communities, demands putting infectious diseases research as the highest priority challenge. These challenges can only be met when coherent strategies aiming at new treatments adhere to a chain of connected pillars, ranging from *basic research funding, translation into drug discovery, diagnosis, prevention*, as well as continuous *efforts in raising public awareness* about threats and proper behavior of each individual.

## Challenges Ahead and Possible Solutions – Making the Impossible Possible?

Although we have been in the post-genomic area for more than 20 years, the burning question why we have not achieved a fundamental understanding of infectious diseases by the onset of the third millennium remains valid, painful and as yet unanswered. A number of explanations are obvious. First, a detailed molecular understanding of basic principles underlying the dynamics of “*host-pathogen*” interactions, which determine the onset, progression and outcome of infections, is missing for many microbial, viral, parasitic and zoonotic pathogens. Even fundamental questions about origin and route of infection, transmission, latency time, progression, host immunity and defense remain open. While such knowledge is pivotal for devising efficient therapies, it is still unavailable owing to the utmost genetic complexity of the individual host, the adaptive potential of pathogens, and of course spatiotemporal disease dynamics. This is especially true for polymicrobial infections, which challenge the host immune defense capacity at all stages by activating pathogen-specific responses and generic inflammatory responses ultimately aimed at pathogen clearance. For instance, we do not know how infection by a single pathogen affects susceptibility to superinfections by other pathogens, including viruses, fungi, parasites or simply triggers the onset of infections by commensal or zoonotic pathogens. There is a lack of understanding when and how the host immune surveillance capacity is saturated in case of co-infections. It is a mystery when self-imposed collateral damage begins or triggers irreversible septic hyperinflammation killing the host rather than the pathogen. Many viral, bacterial, fungal pathogens as well as SARS-CoV-2 often drive lethal septic shocks due to a catastrophic and progressive dysregulation of host immunity. How the complex interplay of epigenetics, environmental factors and genetic predispositions determine pathogen susceptibility is also enigmatic. The lack of rapid and species-specific clinical diagnosis is yet another unmet clinical challenge for many pathogens. Moreover, new mechanistic insights from model systems should be supported by clinical patient data, since results from “*conditioned*” laboratory pathogens are often failing in the clinical *in vivo* validation.

Second, concerted interdisciplinary research efforts are essential for progress yet still in their infancy. It will be critical to identify common denominators in host immunity to various pathogens, the unravel role of trained immunity (Netea et al., 2020), and the translation or validation of basic research into clinical patient settings. We will need to better understand how host immune surveillance shapes and drives the co-evolution of pathogens with the hosts (Woolhouse et al., 2002), especially in the case of opportunistic and zoonotic pathogens. Besides immune defense, other factors may drive the evolution of pathogens, including the increasing chemical diversity of drugs with adverse impact on immunity. Further, dietary habits have significantly changed over the past decades, and environmental parameters such as global warming and climate change, will remain with us for decades to come and severely affect infectious disease susceptibility (Rocklov and Dubrow, 2020). Such shifts may have contributed to the spread of newly emerging pathogens from the environment or wildlife to humans (e.g., influenza from wild birds to poultry). Indeed, climate change may also have triggered the transmission of the thermotolerant fungus *Candida auris* (Lockhart et al., 2017) from environmental niches to birds, and then to becoming a commensal of the human host (Casadevall et al., 2019).

Third, tissue and barrier microbiomes recently emerged as new players in the concert hall of infectious diseases as well as in cancer. There is compelling evidence that dysregulated microbiota contributes or even cause numerous diseases or at least determine susceptibilities (Fiers et al., 2019). However, it remains a conundrum how the dynamic and ultracomplex interplay within and of barrier microbiomes, which encompass thousands of bacterial and fungal species in a metabolic equilibrium, can modulate susceptibility to cancer, infections or autoimmune diseases. Deciphering the impact of dynamic barrier microbiomes on human health, metabolism and well-being provides the next level of as yet unmet challenges. It will require unprecedented interdisciplinary and translational efforts of numerous fields, including a switch from descriptive to mechanistic approaches (Plichta et al., 2019) to unravel how and why dysbalanced immunity-pathogen-microbiota interactions can promote or cause chronic as well as acute diseases (Walter et al., 2020). The underlying complexity will keep scientists busy for decades to come.

Fourth, drug discovery aimed at improving or establishing new anti-infective strategies have always been challenging and outrageously expensive, usually only affordable in developing countries. For decades drugs to treat malaria were prone to MDR and unaffordable for people affected. Pharma companies were reluctant to develop costly antimalarials or vaccines, also because most people affected would not be able to afford treatments. The first malaria vaccine marked a milestone in the treatment of this horrible disease (The Lancet Infectious Diseases, 2019). Pan-anti-infective drugs for groups of pathogens have become less likely, given the persistent threats of MDR. The process from basic research to drug approval sometimes takes more than 15 years, consuming costs up to 2 billion USD/Euros for each new drug. However, this timeline is inconsistent when new infections or pandemics that emerge within a few months, yet no safe treatments are available. And again, COVID-19 set an example, leading to desperation fueling futile drug repurposing efforts (Singh et al., 2020). As for hydroxychloroquine, the hope of therapeutic benefits led politics and science ignore safety issues (Kalra et al., 2020).

Fifth, validated drug safety is another key element required for approval by health authorities. For example, the aging population sometimes use many different drugs on a daily basis, yet predicting drug-drug interactions and adverse effects have remained a clinical challenge, especially for immune cell therapies (Weber et al., 2020). How then can fast-track discovery pipelines be implemented to swiftly react to pandemics without compromising drug safety? Is it only about the amount of input money, global cooperation and new technologies? Maybe yes, perhaps not. Short-cuts in drug discovery process are dangerous (as shown by some examples), and drug safety must never ever be compromised. Drug discovery has to follow a translational, sometimes rocky road that entails trial and error, often requiring animal model systems (Le Magnen et al., 2016), which sometimes fail to translate to human disease. Nonetheless, they are and will remain indispensable for any drug discovery process and to ensure proper translation or not of lead drugs into clinical use. Moreover, pharma industry may have to adopt new strategies for developing anti-infective drugs. Paradigm changes are visible on the horizon. For example, drugging and targeting host immunity can be beneficial for microbial infections, especially for pathogens prone to driving lethal septic immune responses (Cecconi et al., 2018). Modulating or targeting the host immune surveillance rather than killing pathogens may also alleviate or reduce the risk of MDR microbes, a major impediment for some anti-infective approaches especially in ESKAPE pathogens (De Oliveira et al., 2020), as well as in fungal pathogens (Wirnsberger et al., 2016; Berman and Krysan, 2020).

Finally, a better understanding of detrimental or beneficial roles of host immunity in disease outcome will be absolutely essential for successful *precision* or *personalized medicin*e, the new buzzword on the block. The post-genome has been promising personalized medicine strategies for years, yet it has become clear that many challenges, pitfalls and road blocks remain (Willis and Lord, 2015). While some personalized cancer treatments based on patient *omics* data turned out successful (Le Magnen et al., 2016), similar stories for infectious diseases remain scarce. SARS-CoV-2 is again a paradigm example, where the multitude of symptoms (or the lack thereof in some cohorts) is primarily determined by the host tolerance to the virus, making therapies and outcome predictions for such sepsis-prone progressive infections a gamble at best (Singer, 2018). Interestingly enough, many infectious processes seem to follow the laws of game theory, whereby the looser always pays (Chang et al., 2020). At the end, it may simply be the fitness (or tolerance?) of the host or the fitness of the pathogen to resist host defense that determines the outcome (Medzhitov et al., 2012).

## Conclusions and Perspectives

Infectious diseases will undoubtedly persist as permanent and main threats to humanity in the future, especially due to increased longevity that almost always comes at cost due to impaired immunity. One reason among many others is certainly the continuous co-evolution of pathogens with the host immunity, especially in the case of opportunistic and zoonotic pathogens, which is enabling pathogen transmission between different mammalian or animal hosts. How can the research community better address and cope with the challenges ahead of us? Let us briefly discuss a few possible paths to success.

First, a key problem in infectious diseases research is a dramatic lack of public, private as well as corporate funding in many areas. For example, the prevalence and medical relevance of fungal infections is undisputed as they claim about 1.5 million lives every year (Brown et al., 2012), figures that are similar to deaths caused by malaria (Cowman et al., 2016) and tuberculosis (Schrager et al., 2020). Paradoxically, global medical mycology research suffers from a dramatic underfunding in all areas, attracting only a few percent of the total grant support when compared to malaria, tuberculosis or circulatory diseases and cancer (https://www.gaffi.org/). This is also true for many pathogens especially those causing rare infections.

Most infectious diseases are contagious, thus posing global public health threats, which are especially amplified by demographic changes, migration and travel. For instance, tuberculosis, re-emerging measles and most recently COVID-19 are graphic examples. In fact, the COVID-19 pandemic has been telling political decisions makers (and funding agencies) loud and clear that state-of-the art infectious diseases research is a non-negotiable indispensable requirement for any country irrespective of associated costs. Ironically, time will tell if the current increases in funding owing to COVID-19 can be expanded to other infectious diseases. This would also be a positive outcome of the COVID-19 crisis for the people.

Furthermore, it will be mandatory that each country, not continents, should or MUST maintain at least one *national center for infectious disease research*, along the idea of the CDC (https://www.cdc.gov/) or the EDC (https://www.ecdc.europa.eu/en). Epidemiological and public health data must be available in real time to allow for global exchange to ensure and maintain *preparedness* at all times. This will be the only effective way to rapidly recognize and locate disease outbreaks and to identify associated pathogens. Moreover, emergency pandemic plans must be in place in every country, and perpetuated, updated and maintained by health authorities or national centers independent of political partisanship. The “*internet*” became reality and there are no reasons why a “*Virtual Global Infectious Disease Network*” cannot be realized, especially when looking at the heroic efforts of the WHO. We realize that this may be wishful thinking, but it is warranted and urgently required. Governments and health authorities must and can do much better concerning their communication with the public. Political interests must never have priority over public interests concerning health threats, yet we still see on a daily basis how some governments have been using ill-posed strategies or even been hiding facts in dealing with SARS-CoV2.

## The Future of Publishing in Frontiers Microbiology *Infectious Diseases*

The output in infectious diseases research has been exponentially growing or many years (as of November 5, 2020, the search term “infection” yields more than 3,488 million publications in PubMed), yet research is still fragmented into too many small subfields. So, what kind of publications should could we encourage or publish in FMID? The scope definitions make it clear (https://www.frontiersin.org/journals/microbiology/sections/infectious-diseases#about), and perhaps there is not much more to add to this. If one looks at the most cited publications during the past 10 years published in the Infectious Diseases section, most were around the major headlines as discussed for the grand challenges ahead of us, including microbiota dysfunction, microbial drug resistance, host-pathogen interactions for Gram-positive / negative pathogens, viruses like Zika and Ebola, polymicrobial infections, fungal pathogens, exosome function, as well as papers integrating biology and mathematics through *systems infectiology* approaches. Most importantly, we are committed to sustain and further increase the high quality of Frontiers publications, with the main aim to become a prime journal publishing timely research as specified by the scope definitions. Frontiers provides an à* la carte* menu, as the topic *Infectious Diseases* is offered by several distinct Frontiers Journals, sometimes leaving authors wondering as to what the best journal section for their manuscript would be. Perhaps we could consider combining scattered *Infectious Diseases* sections from various Frontiers Journals into a new and unified “*Frontiers in Infectious Diseases*.” This would offer a one-stop shop for authors who wish to publish their most interesting work on various aspects of pathogens. We believe this would be even more attractive to the community, and thus further increase impact and international standing of Frontiers as a leading publication forum in this area.

Finally, and to come to full circle concerning the key question in the title whether we are actually prepared for worst-case scenarios? Sadly enough, the answer is a clear no. We have witnessed how COVID-19 has become a leading global cause of death within just 9 months after its discovery, and all that despite intense health warnings (Cao et al., 2020). Although some laudable efforts and measures were taken by some countries to eradicate COVID-19, the virus keeps spreading around the world. The impression arises that political agenda sometimes prevail over critical health care requirements and improper global communication is a critical problem. However, what is true for SARS-CoV-2, can and will be true for any other pathogen with pandemic potential. It will thus be imperative that governments and health authorities learn the lessons from COVID-19 and implement health care strategies based on scientific facts. If we do not think, act or cooperate globally, we will fail in establishing proper “*preparedness*,” which is essential for both swift and appropriate actions to deal with emerging infectious disease threats.

A famous and iconic scientist from the field, Stanley Falkow, once said “*I never met a microbe I didn't like*” (Falkow, 2008). This so true, since evolution has equipped all pathogens with great many distinct “personalities,” often showing split personalities like Dr. Jekill and Mister Hyde. If we were to better understand and treat the corresponding infections, we must understand their personalities, and to gain such in-depth understanding of pathogenesis, we must like each of them.

## Useful Links for Further Reading

https://www.historyofvaccines.org/timeline/all; https://www.who.int/data/gho/data/themes/topics/causes-of-death; https://www.who.int/en/news-room/fact-sheets/detail/antimicrobial-resistance; https://www.gesundheitsforschung-bmbf.de/en/GlobalAMRHub.php; https://www.gaffi.org/; https://www.cdc.gov/; https://www.ecdc.europa.eu/en.

## Relevant Infectious Diseases Research Topics Links

Our section has contributed over the past decade through a number of attractive Research Topics to obtain better understanding and better knowledge of several infectious diseases, as well as approaches to study and combat them. Some attractive Research Topics and their links were:

- Research Topics on systems biology approaches to decipher **mechanisms of host-pathogen interactions**, and **host immunity** in the course of infectious diseases https://www.frontiersin.org/research-topics/2817/computational-systems-biology-of-pathogen-host-interactions https://www.frontiersin.org/research-topics/5427/integrative-computational-systems-biology-approaches-in-immunology-and-medicine
- Research Topics focusing on diseases of particular importance over the past decade, such as **viral diseases** caused by Zika or Ebola https://www.frontiersin.org/research-topics/4851/zika-virus-research; https://www.frontiersin.org/research-topics/4451/understanding-ebola-a-global-health-challenge
- Research Topics on pathogenomics of **zoonotic diseases** namely brucellosis, which remains one of the most important bacterial zoonotic disease globally https://www.frontiersin.org/research-topics/4920/pathogenomics-of-the-genus-brucella-and-beyond
- Research Topics on **polymicrobial diseases** have been and will be challenging in the future https://www.frontiersin.org/research-topics/3045/polymicrobial-etiologies-of-disease-models-and-perspectives-on-basic-and-clinical-research
- Research Topics on how to address and combat **antimicrobial resistance** https://www.frontiersin.org/research-topics/10012/antimicrobial-resistance-as-a-global-public-health-problem-how-can-we-address-it; https://www.frontiersin.org/research-topics/7705/beyond-antimicrobials-non-traditional-approaches-to-combating-multidrug-resistant-bacteria

## Author Contributions

KK and AC conceived and wrote this manuscript. All authors contributed to the article and approved the submitted version.

## Conflict of Interest

The authors declare that the research was conducted in the absence of any commercial or financial relationships that could be construed as a potential conflict of interest.
